# GPS Trajectory Completion Using End-to-End Bidirectional Convolutional Recurrent Encoder-Decoder Architecture with Attention Mechanism

**DOI:** 10.3390/s20185143

**Published:** 2020-09-09

**Authors:** Asif Nawaz, Zhiqiu Huang, Senzhang Wang, Azeem Akbar, Hussain AlSalman, Abdu Gumaei

**Affiliations:** 1 Department of Computer Science and Technology, Nanjing University of Aeronautics and Astronautics, Nanjing 210016, China; zqhuang@nuaa.edu.cn (Z.H.); szwang@nuaa.edu.cn (S.W.); L1600308@cqu.edu.cn (A.A.); 2 Key Laboratory of Safety-Critical Software, Nanjing University of Aeronautics and Astronautics, Ministry of Industry and Information Technology, Nanjing 211106, China; 3 Collaborative Innovation Center of Novel Software Technology and Industrialization, Nanjing 210093, China; 4 Department of Computer Science, College of Computer and Information Sciences, King Saud University, Riyadh 11543, Saudi Arabia; halsalman@ksu.edu.sa

**Keywords:** GPS trajectory, ConvLSTM, encoder-decoder, attention, trajectory completion

## Abstract

GPS datasets in the big data regime provide rich contextual information that enable efficient implementation of advanced features such as navigation, tracking, and security in urban computing systems. Understanding the hidden patterns in large amount of GPS data is critically important in ubiquitous computing. The quality of GPS data is the fundamental key problem to produce high quality results. In real world applications, certain GPS trajectories are sparse and incomplete; this increases the complexity of inference algorithms. Few of existing studies have tried to address this problem using complicated algorithms that are based on conventional heuristics; this requires extensive domain knowledge of underlying applications. Our contribution in this paper are two-fold. First, we proposed deep learning based bidirectional convolutional recurrent encoder-decoder architecture to generate the missing points of GPS trajectories over occupancy grid-map. Second, we interfaced attention mechanism between enconder and decoder, that further enhance the performance of our model. We have performed the experiments on widely used Microsoft geolife trajectory dataset, and perform the experiments over multiple level of grid resolutions and multiple lengths of missing GPS segments. Our proposed model achieved better results in terms of average displacement error as compared to the state-of-the-art benchmark methods.

## 1. Introduction

The Internet of things (IOT) is a technological revolution that rapidly reshapes the real society through ubiquitous sensor devices [1]. As more and more sensors are deployed, the amount of data collected is also significantly improved, which makes the data mining technology in the Internet of things play an increasingly important role [2]. The data collected in an IOT environment can be used in many different application ranging from natural language processing [3], health care systems [4,5,6], to transportation systems [7,8,9]. Trajectory data is a kind of specific data that can be collected frequently in IOT environment data, which has attracted extensive attention in recent years. Global Positioning Systems (GPS) is a comprehensive, high navigation satellite positioning system that record spatiotemporal information of users/vehicles while moving in a traffic network. Large-scale GPS data is collected around the world with higher accuracy has grown exponentially over the past few decades using the most advanced Global Navigation Satellite Systems (GNSS). In addition, most of the modern positioning devices are equipped with modern GNSS like Galileo, GLONASS [10] and so forth. Therefore, GPS based system becoming more demanding, as GPS data is easily available and anonymously produced by many positioning devices with higher accuracy. The advances of GPS-enabled devices and mobile computing techniques enabling human to record their trajectories, that represents human behaviors and travel related preferences. Devices equipped with these technologies (e.g., drivers’ smart phones) can track the locations of moving objects’ to create their trajectory data. The study of GPS trajectory data mining leads to a diverse set of behavioral applications including anomaly detection [11], learning significant locations [12], Traffic prediction [13], location based activity recognition [14], and traffic congestion estimation [15]. All such applications require analysis of higher quality GPS data that is the focus of our study.

A GPS trajectory is a connected sequence of GPS points that are chronologically ordered, and represented by $p_{1}\to p_{2}\to \cdots \to p_{n}$. Each GPS point $p_{i}$, is denoted as (x,y,t), where x,y, and *t* are latitude, longitude, and timestamp respectively [16]. These trajectories provide us with unprecedented information to understand the movement pattern of the objects and their positions, and facilitates a wide range of applications including location-based social networks (LBSN) [17], intelligent transportation systems [18], urban computing [19] and many more. Under these circumstances, the research on GPS trajectory data mining is of great significance, which has attracted attention in computer science, sociology, geography and many other fields [20]. The process of GPS data collection suffers from few major drawbacks of missing data that usually occurs because of inaccurate signals, device errors and data privacy issues; that in result leads to missing GPS points. For example, the whole trajectory is $\left(l_{1},l_{2},l_{3},l_{4},l_{5},l_{6}\right)$. However, due the the privacy issue, the users only report their locations of $\left(l_{1},l_{3},l_{6}\right)$. This is because without the permission of the users, we cannot get the location information. Our goal is to complete the whole trajectory based on the partial trajectory reported by the user so that we can better provide her/him service like Point of Interest (POI) recommendation when the user is at the location $\left(l_{2},l_{4},l_{5}\right)$. Higher quality GPS data is important to produce the results with higher accuracy. Common techniques used to deal with missing data is to replace the missing data with some values or ignore them, but it does not provide reliable estimation of the ground truth. Therefore, it becomes very important to innovate the techniques to reproduce missing GPS data.

The process of imputing missing parts of the trajectories involves to find the patterns in similar trajectories using multiple features. The trajectory completion task is quite challenging as it requires to learn complex traffic patterns in big cities environment [21,22]. Many existing studies like References [23,24,25] try to derive the complex trajectory prediction algorithms using sophisticated behavior modeling, that requires the system designers to do in-depth model optimization, and can only model the limited set of patterns. Traditional shallow methods like probabilistic models, nearest neighbors are not able to effectively capture non-linear relationships in the data. It also requires large amount of time and human expertise to hand-engineer the most representative features for prediction task. Our approach is data-driven and easier to understand, that automatically learns complex behaviors and patterns from large amount of GPS trajectories using deep neural network models. Based on these patterns and behaviors we can predict the missing parts of the trajectories.

GPS trajectory completion is kind of sequence to sequence prediction problem. Many challenging sequence to sequence prediction tasks were solved using state of the art deep learning methods in recent decade like machine translation, travel route predictions, weather forecasting and so forth [26]. Based on the success of deep learning techniques in recent era, we choose deep net based architecture to solve our problem of GPS trajectory completion only based on GPS data. A few studies, like References [14,27], use data from other sensors like accelerometer, gyroscope, WiFi with the GPS data, and similarly few studies makes use of GIS information with the GPS data [28] for trajectory mining tasks. It is not always feasible to carry multiple sensors, or WiFi is not necessarily available at all locations. GIS data is also not available for all the cities and locations, or may change and updated from time to time that is difficult to incorporate with the model. Therefore, it becomes more important to make the system that is only based on GPS data. Our study only uses the GPS data without the support of data from other sensors and without the support of underlying maps data, that also makes the problem more general and challenging.

Following are the core contributions of our work.

- To solve the trajectory completion problem, we for the first time propose deep learning based convolution recurrent encoder decoder architecture, to predict the complete sequence of GPS points from partial input of GPS trajectory points on occupancy grid map.
- Due to the spatiotemporal nature of GPS data, we used Convolutional Long Short Term Memory (ConvLSTM) based encoder decoder architecture using attention mechanism.
- To interpret the missing points in a sequence, it is important to know the information from both past and future timesteps. For this purpose, we make use of bidirectional encoder to encode the missing trajectory.
- Our model can effectively integrate global features and auxiliary information that support the encoder-decoder model for trajectory completion task with high accuracy.

The rest of the paper is organized as follows—we describe the related work in Section 2. In Section 3, we describe in detail our proposed framework for the completion of GPS trajectories. Experimental results are provided in Section 4, and the paper is concluded in Section 5.

## 2. Related Work

Many GPS devices prone to failures that results in missing GPS data. The missing data is characterized for longer duration during which no data are observed [29]. Few studies proposed the algorithms to predict missing parts of GPS trajectories that are based on statistical techniques, human knowledge and expertise. Zheng et al. [23] infers the missing part of a sparse GPS trajectory by comparing with the sequence of reference trajectories generated from historical trajectories. Shen et al. [29] proposed the algorithm for trajectory completion but do not achieved the desired results. Goren-Bar et al. [30] uses the path approximation algorithm, that aggregates the points in multiple trajectories belonging to the same road, and finds the principle curve for each group. Similarly, References [25,31,32] try to infer the missing parts of trajectory using the knowledge learned from historical trajectories. All these studies either requires information of road network, or require domain expertise and human expertise to identify the patterns in similar historical trajectories. Real world road networks updating regularly, therefore learning based only using GPS data is more challenging. The conventional approaches also do not effectively capture the non-linear relationship in the data to complete the missing parts of trajectories. Therefore, deep learning based models are most suitable and state of the art methods to do all these tasks by automatic feature learning.

Deep learning methods are state of the art widely used techniques in many different applications ranging from medical image processing, transportation problems, urban computing and so forth. These approaches are also heavily used in similar problems like image completion [33], machine translation [34], travel route planning [35] and so forth, and achieved outstanding results. Xiaoguang et al. [33] proposes volumetric Convolution Neural Network (CNN) encoder decoder architecture to identify the missing parts of the 3D shapes. Kim et al. [36] uses the Recurrent Neural Network (RNN) based Long Short Term Memory (LSTM) architecture to predict the future locations of the trajectory over occupancy grid map. Wang et al. [37] represents the GPS trajectory as a 2D image by dividing the whole geographical space into two dimensional grid and each grid cell is represented as one pixel. Wu et al. [34] proposed end to end LSTM encoder decoder architecture for sequence learning in neural machine translation. Inspired from the tasks of image completion, trajectory representation on grid map and sequence to sequence based route prediction and machine translation; we propose deep learning based methodology that uses the knowledge from all above mentioned tasks to infer the missing part of GPS trajectories. Our effort is to exploit the strengths of deep learning methods that identifies the hidden patterns automatically without using the human knowledge and domain expertise in order to complete the missing parts of trajectories over occupancy grid map. Based on sequential time series nature of GPS trajectories, we used recurrent neural network architecture to exploit the hidden patterns in spatiotemporal time series GPS data. The input features in the deep learning architecture is the original object, rather than the set of hand engineered features, such as the GPS segment. The fundamental characteristic of deep learning technology is to encode the original and low-level features of an object, such as the GPS point into multiple levels of efficient and high-level features using the set of operations in hidden layers [38]. This is the most significant characteristic of deep learning techniques that differentiates them from conventional machine learning algorithms. The deep features or the encoded representations of the set of input features are most effective and generated automatically instead of involving human efforts as in conventional machine learning approaches [11]. It is important to note that the features learned in the last layer have the same role as handcrafted features, which are entered into activation functions (for example, SVM or softmax) to calculate the class probabilities [39].

The great diversity of neural net architectures is feed forward neural network, convolution neural networks and recurrent neural networks [40]. Among all these architectures LSTM is a special case of RNN, that has proven to be more successful on sequential data tasks or time series prediction such as machine translation [34]. This is mainly due to their ability to memorize long term dependencies, which is achieved by taking into account previous information for further predictions. Learning of sequential data continues to be a fundamental task and a challenge in pattern recognition [41]. GPS data is spatiotemporal in nature; the GPS points in a trajectory are both spatially and temporally correlated. CNN architectures achieved great success to learn patterns in spatial data, whereas recurrent nets become more successful in predicting sequential time series [42]. Due to the spatiotemporal nature of GPS data, we make use of ConvLSTM architecture [43], which exploits the strengths of both CNN and LSTM in a single architecture, thus highly capable to model the spatiotemporal time series data. Due to variable length of input and output sequences, we make use of encoder decoder architecture using attention mechanism inspiring from the task machine translation [34]. In addition, encoder decoder architecture of our approach is also supplied with additional auxiliary information that remains the same throughout all time steps [42], and the global features extracted in parallel using convolutional layers that further enhances the performance of our proposed approach.

## 3. Approach

We start this section with the description of dataset that we used to train our model. In the next part of this section, we describe the preprocess of raw GPS data to prepare the data for our model. This is followed by problem definition and ConvLSTM architecture to model the GPS trajectories. Next section describes the broader overview of our architecture, which is followed by detailed description of encoder-decoder architecture for our model. The last subsection of this section given the detailed description of our input features.

### 3.1. Data Description

**Figure 1:**
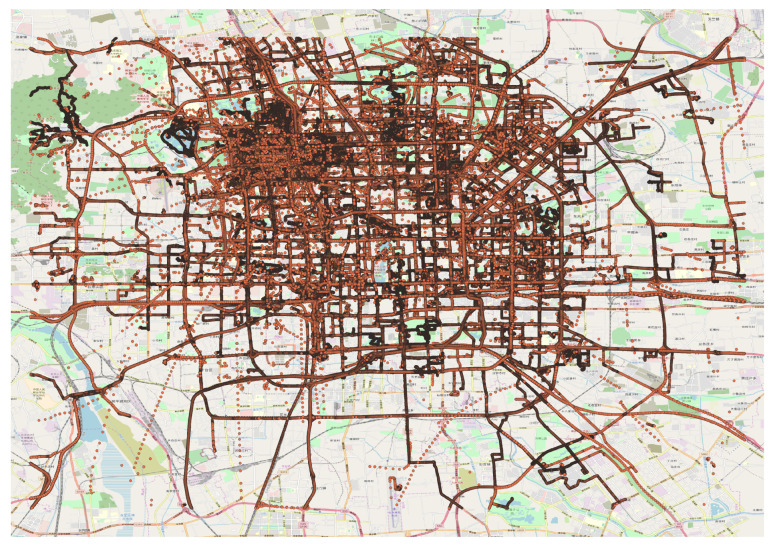
Microsoft Geolife trajectory dataset in Beijing.

We used Microsoft geolife trajectory dataset [12,44,45] to evaluate the performance of our proposed approach. Geolife trajectory dataset is widely used benchmark dataset used in many trajectory mining tasks. The GPS data was collected by 182 users in 30 different cities of China that spanned over the time period of 5 years from April 2007 to August 2012. The dataset contains 17621 trajectories. Most of the dataset is recorded in Beijing by many volunteer GPS loggers. Therefore, we used the data in Beijing city that has complex traffic patterns with dense representation of GPS points as shown in Figure 1.

### 3.2. Data Preprocessing

**Figure 2:**
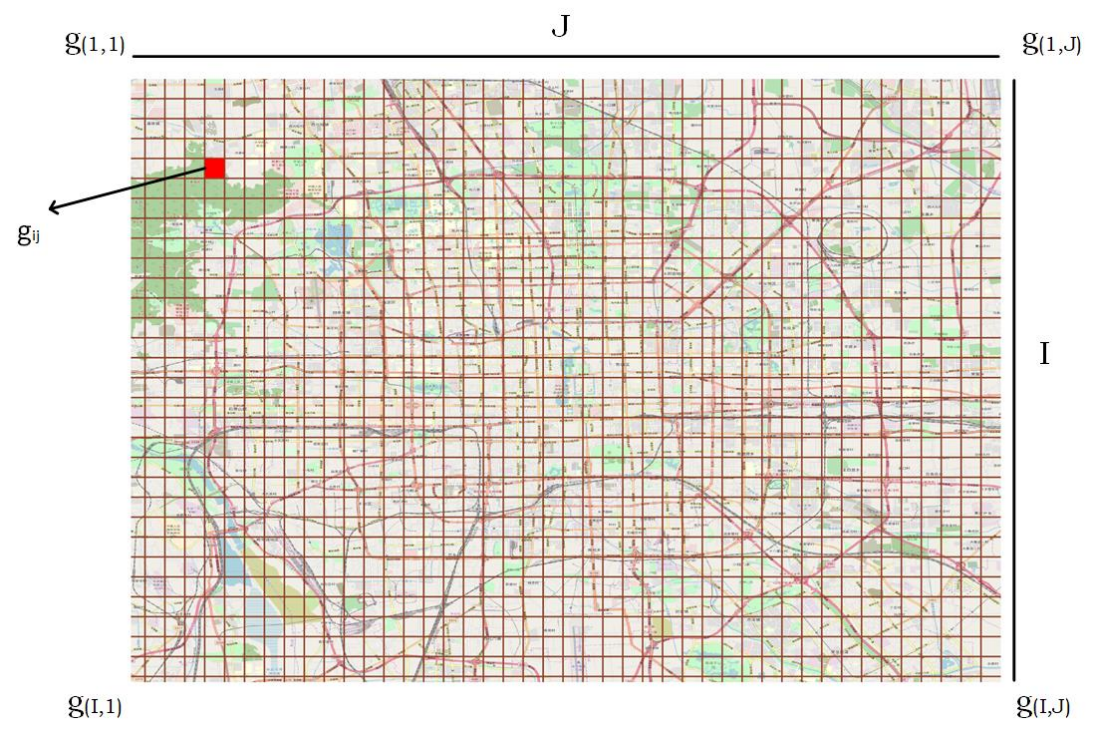
Map grid representation.

GPS trajectory is the sequence of points ordered with respect to time. We model all trajectories of Microsoft geolife trajectories [45] over two-dimensional occupancy grid-map, which is achieved by dividing whole geographical space into two-dimensional grid, where all grid cells are equal size as shown in Figure 2.

In Figure 2, *I* represents the number of rows, whereas *J* represent the total number of columns in two-dimensional grid. $g_{ij}$ represents the specific grid position of a GPS point in grid G. Thus all GPS points are represented with its respective grid positions in a two-dimensional grid. Similarly, all trajectories and segments are represented with the sequence of grid positions in a two-dimensional equal sized grid.

Neural networks require fixed length input in their models, so we need certain operations on GPS trajectories to divide all trajectories in Microsoft geolife trajectory dataset into fixed length input segments. The first step is to divide all trajectories into trips, if the time duration between two consecutive points exceeds certain time delta threshold. In next step all trips are further subdivided into fixed length input segments each of size n. The size of last segment may be less than the threshold size of input segment, in that case we pad zero values to make the segment length equals all others input segments. Neural networks perform better if trained with more data. For this purpose, we used moving window approach to create more input samples, this approach is known as data augmentation; which is the useful approach to achieve best performance of deep neural net architecture in order to learn more complex patterns and behaviors in large set of data [46]. In addition to moving window sampling, we also used reversed order input sequences to train the model, that further augments the data and to learn the more complex patterns. Next ground truth trajectories are prepared. For the ground truth, we assume that some locations of a trajectory are missing and removed from the sample trajectories. Then we try to complete the removed locations and compare the predicted trajectories with the ground truth trajectories. In the following subsections we describe problem description, discuss with detailed description of our model architecture and our input features.

### 3.3. Problem Definition

A GPS point is represented by 2D coordinate $\left(x_{t},y_{t}\right)$ on a grid *G*, where *x* and *y* are position of an object at time instance *t*. Given the observed locations of moving object at time t=1 to t=m−1 and t=n+1 to t=s. Our aim is to predict the positions of objects from time t=m to t=n. Thus, given the incomplete trajectory $X_{incomp}=\left(x_{1},y_{1}\right),\left(x_{2},y_{2}\right)\ldots (x_{m-1},y_{m-1)}\ldots \left(x_{m+1},y_{n+1}\right)\ldots \ldots ,\left(x_{s},y_{s}\right)$, we aim to predict the missing part $X_{miss}=\left(x_{m},y_{m}\right),\left(x_{m+1},y_{m+1}\right)\ldots \left(x_{n-1},y_{n-1}\right),\left(x_{n},y_{n}\right)$ of length *l* in the completed trajectory $X_{comp}=\left(x_{1},y_{1}\right),\left(x_{2},y_{2}\right)\ldots .\left(x_{s},y_{s}\right)$, where *l* is the length of missing sub-trajectory *X*, and l=n−m.

### 3.4. Convolutional Long Short Term Memory (ConvLSTM)

**Figure 3:**
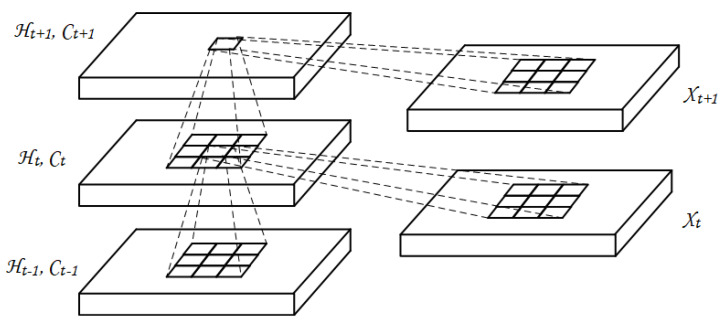
Inner structure of ConvLSTM.

One of the limitation in FC-LSTM (Fully Connected Long Short Term Memory) is the loss of spatial information while processing the spatiotemporal data, as the input to LSTM must be transformed into a one-dimensional vector to process the data. ConvLSTM is the powerful variation of LSTM, that performs the convolution operation on input sub-sequences within the LSTM unit, thus able to retain both spatial and temporal information. This approached has proved to be more successful in time series classification and multi-step forecasting. The ConvLSTM architecture determines the future state of the grid cell using the input and past states of its local neighbors. This is achieved using the convolution operation in state-to-state and input-to-state transition as shown in Figure 3. Equations (1) to (5) below are the key equations of ConvLSTM, in which ‘∗’ represents the convolution operation and ‘∘’ represents Hamadard product [43].

(1) $$i_{t}=\sigma \left(W_{xi}*X_{t}+W_{hi}*H_{t-1}+W_{ci}\circ C_{t-1}+b_{i}\right)$$

(2) $$f_{t}=\sigma \left(W_{xf}*X_{t}+W_{hf}*H_{t-1}+W_{cf}\circ C_{t-1}+b_{f}\right)$$

(3) $$C_{t}=f_{t}\circ C_{t-1}+i_{t}\circ tanh\left(W_{xc}*X_{t}+W_{hc}*H_{t-1}+b_{c}\right)$$

(4) $$o_{t}=\sigma \left(W_{x}o*X_{t}+W_{h}o*H(t-1\right)+W_{c}o\circ C_{t}+b_{o})$$

(5) $$H_{t}=o_{t}\circ \tanh\left(C_{t}\right)$$

where $X_{t}$ is the input segment at time *t*. $i_{t}$ is the input gate at time *t*, $f_{t}$ is forget gate, $c_{t}$ is cell state, $o_{t}$ is output gate and $h_{t}$ is the hidden state at time *t*. Similarly, $i_{t-1}$ is the input gate, $f_{t-1}$ is forget gate, $c_{t-1}$ is cell state, $o_{t-1}$ is output gate and $h_{t-1}$ is the hidden state at time t−1. The gating vectors determines what information to update, what to forget and what to pass through by the output gate. σ and tanh are sigmoid and hyperbolic tangent functions respectively; similarly, *W* and *b* are the weights and bias vectors respectively.

Figure 3 is the basic representation of ConvLSTM; both input and inner representations are two dimensional in case of one channel, thus enabling the model to encode both the temporal and spatial correlations [43]. From Figure 3, it can be seen that the feature map or the cell state at time instance *t* is produced by applying the convolutional filter on hidden state vector with its weight vector at previous time step t−1 (state to state transition), and similarly convolving the input vector *X* with its weight vector at the current time step *t* (input to state transition) given in Equation (3).

### 3.5. Model Description

**Figure 4:**
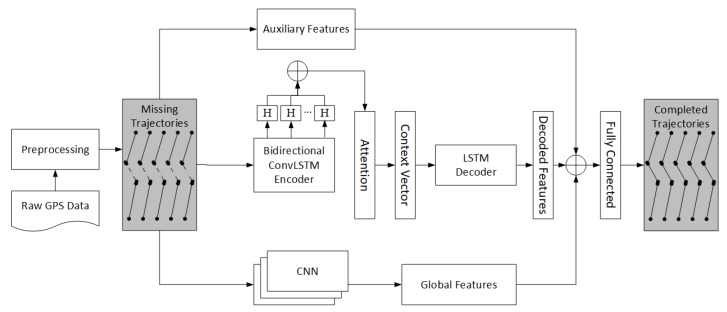
Proposed approach architecture for trajectory completion.

Our model is mainly based on deep learning based encoder decoder architecture that proved to be more successful in sequence prediction problems [34]. Encoder module is responsible to encapsulate the information of entire input sequence into a fixed length context vector, which is then passed to the decoder, that is responsible for stepping through the output time steps while reading the context vector. Figure 4 elaborates our proposed architecture for trajectory completion task.

The input to the model are raw GPS data, which is first preprocessed to prepare the input to the model. The input to the model are preprocessed trajectories, which is the sequence of GPS points. The details of preprocessing steps are given at the start of this section. The prediction model is trained using the set of incomplete trajectories as input and completed trajectories as output of the model. Once the model is trained, it can be used to predict the missing parts of the sequence of missing observations. The architecture is trained into three parallel steps as shown in Figure 4. In first step, basic input features like velocity, acceleration, jerk are feed into ConvLSTM based encoder decoder architecture, that produces the decoded features. In addition to the decoded features, auxiliary features are extracted directly from input data. Global features are also extracted in parallel to auxiliary and decoded features. The details of our encoder decoder architecture, and the input features are given in following sections. Decoded features are merged with auxiliary and global features and passed to fully connected layer, that predict the probability distributions of GPS points for all time steps. We used the widely used beam search algorithm [34] to sample the output sequence of GPS points with highest probability. To validate our model, we used mean squared error loss function J during the training using the Equation (6) given below.

(6) $$Loss\left(y,\hat{y}\right)=\frac{1}{N}\sum_{i=1}^{N}{\left(y_{i}-{\hat{y}}_{i}\right)}^{2}$$

### 3.6. Encoder Decoder Architecture Using Attention Mechanism

In order to predict the missing sequence of GPS points in between the trajectory, this is necessary to know the information both from previous and future timesteps. For this purpose, we make use of bidirectional encoder so that the movement patterns in both directions can be captured [47]. Due to the spatiotemporal nature of GPS trajectory data, we make use of ConvLSTM architecture [43] in our model that is able to deal with both spatial and temporal dependencies in the data. To input the segment into ConvLSTM architecture, first the segment is divided into multiple windows (sub-segments) of equal lengths. The total number of sub-segments are considered as the total number of timesteps to deal with temporal relationships in ConvLSTM cell. One dimensional convolutions filter of size n is applied in each window to deal with spatial characteristics and to produce the feature map at the next time step. In a conventional encoder decoder architecture, encoder only passes the state from last cell to the decoder. To address this limitation of simple LSTM based encoder, we used attention layer as an interface between encoder and decoder for latent representation of the input features, that keeps track of all previous states with different weights [34]. Attention provides the richer context from encoder to the decoder and the learning mechanism in which decoder can learn at each time step; where to pay the attention on encoded vector to predict the output sequence. It helps the architecture to memorize long term dependencies between the observations at different time steps and able to learn more complex patterns in sequence of GPS points. The weighted context vector is produced as the output of the encoder, that enables the decoder to predict the subsequent GPS points in a sequence. Context vector here is the representation of input features at different time steps weighted by their attention weights. Attention weights here represents how much attention to pay to the specific region to the inputs at specific time steps, and sum of attention weights equals to 1. The attention weights are computed using the following set of Equations (7) to (10) given below.

(7) $$e^{t}=\left[s_{t}h_{1},\ldots \ldots ,s_{t}h_{N}\right]$$

(8) $$\alpha^{t}=softmax\left(e^{t}\right)$$

(9) $$a_{t}=\sum_{i=1}^{N}\alpha_{i}^{t}h_{i}$$

(10) $$c=[a_{t},s_{t}]$$

**Figure 5:**
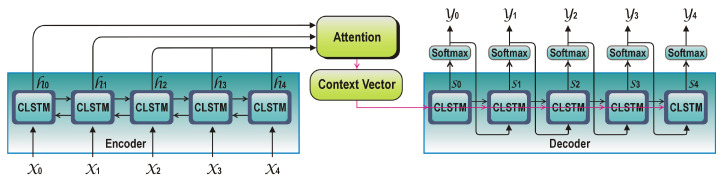
Bidirectional convolution recurrent encoder decoder architecture using attention mechanism.

In a sequence to sequence prediction problems, each hidden state $s_{t}$ of the decoder attends to all hidden states of the encoder $h_{1},h_{2},\ldots ,h_{N}$. At each timestep of the decoder, there is a direct connection with the encoder but focused on different parts of the input GPS segment. In Equation (7), attention score $e_{t}$ is calculated by taking the dot product of current hidden state of the decoder at time step *t* with all the hidden states of the encoder. softmax function is used to normalize all attention scores; that generates the attention distribution $\alpha^{t}$ at time step *t*, shown in Equation (8). Attention weights $a_{t}$ given in Equation (9) are computed by taking the weighted sum of attention distribution at time *t* with the encoder’s hidden states. In Equation (10), attention output $a_{t}$ is concatenated with the decoder’s current hidden state $s_{t}$, that becomes the context vector *c* to proceed with predicting next points at subsequent time steps. The functioning of encoder decoder part of our model is shown in Figure 5.

The decoder processes the context vector using predicted output at previous timestep to produce the probabilities of next GPS points. The output of the decoder is the hidden representation of decoded features.

### 3.7. Input Features

In this subsection, we give detailed description of the features used by our model. We categorized the input features into three categories, that is, auxiliary features, local features and global features. Details of these features are given below.

#### 3.7.1. Auxiliary Features

The movement behaviors of any moving object depends on some external features or auxiliary features. For example, behavior of traffic may vary during peak hours and non-peak hours, or the movement patterns of moving object may vary on weekdays and non-weekdays. These features are important to learn distinct patterns. These features are extracted directly from the input data, and remains the same throughout all timesteps of input samples or among different points of GPS trajectories. Instead of passing these features as encoder’s input, we bypass these features and merge directly with the decoded features as shown in Figure 4. Bypassing these features reduces model complexity to avoid hidden layer operations by the encoder module. In our study, we used two auxiliary features. One is weekday, its value is either weekday or weekend, based on day of the week. Other auxiliary feature is peak time; its value is either busy or idle based on time of the day.

#### 3.7.2. Local Features

These are the features that relates to the GPS point like velocity, speed, acceleration and so forth. Using the basic information of latitude, longitude and timestamp of each GPS point of a segment, we compute certain hand-crafted motion features of all GPS points like distance, time, velocity, acceleration, acceleration rate and heading change rate given in the study [44]. As the feature like velocity, acceleration, accelerate rate and bearing rate all can be derived from distance and time, so we do not consider distance and time features as input to the encoder to avoid the problem of multicollinearity, as this issue degrades the quality of automatic feature learning in deep learning models [39]. In addition to these features, we also input another feature grid index, that represents the position of a GPS point in a two dimensional grid. This is achieved by dividing the whole geographical region in a 2D grid *G*, where all grid cell sizes are of same size as shown in Figure 2. Each grid cell is represented by a row and column number in *G*. The two attributes rows and columns are converted to a single attribute grid index to represent the trajectory using one-dimensional sequence of numbers (grid indices) using the Equation (11) given below.

(11) $$\begin{array}{c} g_{i}=\left(i-1\right)\times J+j \end{array}$$

where *i* and *j* are the row and column number of a GPS point in two-dimensional grid *G*. *J* is the total number of columns in two dimensional grid *G*.

#### 3.7.3. Global Features

CNN is useful architecture using large filter sizes in order to extract the global features [33]. These features cover the wider geographical context of a trajectory in our context. Global features are the correlations among neighboring grid cells, representing the movement probabilities between different regions based on the densities of GPS points in the grid cells. Global features are obtained by training another convolutional network in parallel to encoder decoder network as shown in Figure 4. The global features learn the movement patterns of GPS trajectories between different parts of the geographical region. The input to the CNN is the position of a GPS point in 2D grid along with grid densities. Input feature helps to derive deep probabilistic latent feature that describes the importance of the regions in a geographical space. The deep global features are merged at later stage with the output of decoder and auxiliary information to predict the missing sequence of GPS points in a trajectory.

## 4. Experiments

In this section, we discuss about the results of our experiments. We start this section with the description of evaluation metric to evaluate the effectiveness of our approach. We then describe the configurations of our architecture. At the end, we explain the results of our experiments. We used QGIS version 3.10.2-A for the visualization and analysis of GPS data. We used python v.3.7 for the preparation of input data, and used the python based deep learning library keras version 2.2.4 to implement our proposed architecture.

### 4.1. Evaluation Metric

For our experiments, we used Average Displacement Error (ADE) as the benchmark metric of the trajnet challenge [48] as our evaluation metric, which is the measure of euclidean distance between the actual trajectory location and predicted trajectory location over the occupancy grid map for all the trajectories, as shown in Equation (12).

(12) $$ADE=\frac{\sum_{j=1}^{N}\frac{\sum_{i=1}^{n}{\left({\hat{x}}_{i}^{j}-x_{i}^{j}\right)}^{2}-{\left({\hat{y}}_{i}^{j}-y_{i}^{j}\right)}^{2}}{n}}{N}$$

where $\left(\hat{x},\hat{y}\right)$ are coordinates of predicted trajectories and (x,y) are coordinates of ground truth trajectories. *n* is the total number of points in a segment and *N* is the total number of segments.

### 4.2. Training Setup

The geographical space is divided into two dimensional grid of equal sizes. To evaluate the performance of our model, we used different resolution grids from 2 sq. meter to 10 sq. meter for each grid cell. If the predicted point and ground truth both lies in same grid cell, it is assumed to be correctly identified. All GPS tracks are divided into trips if the time duration between two consecutive GPS points is greater than 20 min [49]. All trips are divided into equal size segments of length 200 GPS points, that is the median length of all segments, these segments are the input to the model. From the dataset, we take the set of complete GPS trajectories, from which we omit the portion of segment ranging from 10 GPS points to 30 GPS points; we consider these incomplete trajectories as our ground truth. The value of auxiliary feature peak time is set to true if the time of the day is between 7:00 a.m. to 10:00 a.m. and from 4:00 p.m. to 7:00 p.m., and false otherwise [50]. For the weekday, its value is true if the day of the week is from Monday to Friday and false otherwise. In order to have more data samples to support data augmentation and learn the optimized parameters, the input samples are created using moving window strategy with the stride of 25.

**Figure 6:**
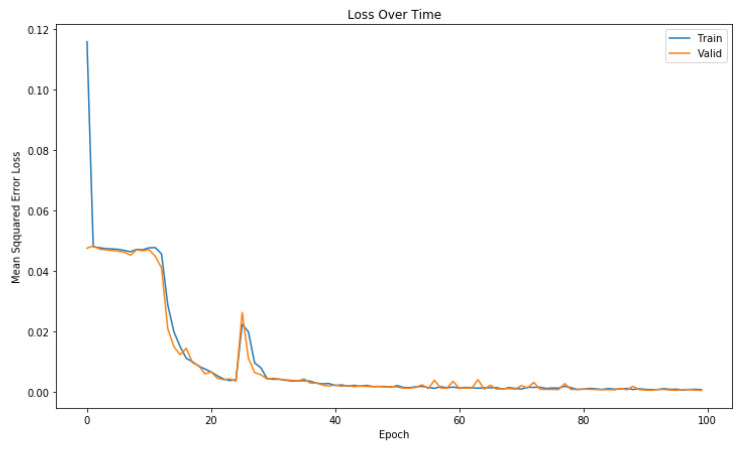
Training vs. validation loss graph.

We observed the performance of our proposed model using different settings of hyper-parameters. The optimal performance is achieved using the following settings in our architecture. We used 2 ConvLSTM layers for encoder, one in each direction; and also the 2 ConvLSTM layers in decoder module. All layers in encoder and decoder uses 200 units. We used 64 filters in first layer of ConvLSTM in encoder and 128 filters are used in the second layer. 1 × 3 filter size is used for one-dimensional row vector to learn local features. Similarly same configuration settings of number of filters and filter sizes are used for decoder module. The original input segment need to be transformed into 3D tensor to input into ConvLSTM encoder. For this purpose; the full length input segment of size 200 is divided into 8 timesteps; each time step contains 25 columns for 1 row vector. For sequence prediction problems, the network is optimized using mean square error loss function, which is the distance between predicted point and the ground truth actual point over all timesteps. To learn global features, we used 2 CNN layers with filter size of 1 × 7 and the stride of 1. The model is trained on 100 epochs with efficient adam optimizer ofstochastic gradient descent using the default configuration, in order to optimize model weights. SoftMax layer at the end uses 200 neurons, one for each output GPS point of a sequence in trajectory segment. We also used 20% dropout value is used to avoid overfitting of the model. Beam search size is set to 5. Model is trained using the set of batches each of size 256 samples. 80 percent of total data set is used for training purpose, out of which 10 percent is dedicated for validation purpose, Whereas the rest 20 percent used as the test set. Figure 6 shows the training and validation loss for 10 m grid resolution by training the model at 100 epochs. It can be seen from Figure 6 that the learning of our model improves and converges quickly using the mean square error loss value, and both training and validation losses decrease and converged reasonably well over 100 epochs.

### 4.3. Results and Discussion

This section provides the precise description, interpretation of experimental results and the drawn conclusion. We compare the performance of our method in two different ways. One by comparing with different benchmark inference methods, and other is by analyzing the performance of our approach by predicting varying lengths of missing timesteps.

#### 4.3.1. Comparison with State of the Art Methods

To evaluate the effectiveness of our approach, this is necessary to make comparison with different state-of-the-art approaches for trajectory completion task. It can be seen from Table 1 that our encoder decoder architecture performs better in terms of average displacement error as compared to all benchmark models for all level of grid resolutions from 2 m to 10 m. Table 1 shows that our proposed approach performs better to predicts 10 missing points to complete the trajectory

The results in Table 1 shows that our method provides the stable results for different resolution of grids from 10 m to 2 m using the ADE metric. The ADE value represents how far is the predicted point from its actual position in terms of number of grid cells. It can be seen from Table 1 that LSTM Encoder/Decoder gives better performance in terms of average displacement error among all other benchmark models. It can also be seen that performance of ConvLSTM encoder decoder has significantly improved as compared to LSTM encoder decoder architecture. We further analyzed the impact of attention mechanism, it can be observed that ADE loss has further decreased using the attention mechanism in ConvLSTM encoder decoder architecture. We also analyzed the impact of auxiliary and global features (AuxGlobal) in our proposed architecture, the ADE loss is further decreased when AuxGlobal features are incorporated with attention model. Bidirectional ConvLSTM encoder decoder using attention mechanism and AuxGlobal features is the final configuration of our architecture, and the overall ADE loss has further decreased using the final configuration. We can also observe from Table 1, that 10 m resolution grid occurs less error value. The reason for this is that, the 10 m grid cell covers wider geographical space, and there is high probability that the small prediction error of the predicted point lies in the same grid cell as ground truth. Whereas it is less likely in case of 2 m resolution grid because 2 m grid cover limited geographical space and there are high chances that the very small prediction error of the predicted point may be identified in other grid cell. The high error value in high resolution grid (like 2 m grid) is because of the noise in observed GPS trajectories.

#### 4.3.2. Evaluation of Proposed Method

We further investigate the performance of our final configuration of encoder decoder model to interpret different missing lengths of sub segments, and it can be seen that our approach has proved the acceptable performance. We evaluate the performance of our method for different lengths of missing points; that is, from 10 to 30 missing timesteps of a GPS segment. Table 2 shows the comparison of ADE loss performance of our proposed convolutional recurrent encoder decoder model to predict different lengths of missing timesteps.

The results in Table 2 show that the ADE loss slightly increases with the increasing length of missing sub segments (top to bottom). It can be seen that the value of ADE loss increases with the increased grid resolution (from left to right), especially in high resolution grids like (2 m grid segment cell). The overall loss can be reduced further by training the architecture on large dataset, that is the key major factor in neural nets to optimize the parameters.

We have evaluated our approach on different grid resolutions ranging from 2 m to 10 m grid size. The geolife trajectory dataset have densed representation with a good sampling rate from 2 m to 5 m. The key property of deep learning based methods that it works well on big data set with densed representation of the data. Based on such characteristics of data and our modeling approach, our model can generalize well on all other dataset having densed representation of GPS data and to have a good sampling rate.

## 5. Conclusions and Future Work

In this paper, we presented the study for the completion of missing sub-trajectory over occupancy grid map. We come across the unusual shortcomings in existing works and proposed the solution to solve the trajectory completion problem using state of the art deep learning methodology. Our proposed methodology bidirectional convolutional recurrent encoder decoder architecture shown significant improvement over the benchmark models in terms of average displacement error loss function. The results are further improved using attention mechanism and AuxGlobal features with our basic ConvLSTM based encoder decoder model. These results can be improved further, if trained on a bigger dataset in order to learn more complex and distinct patterns. In this work, we only predict the estimated position of a GPS point over occupancy grid map. Our study considers more general and common scenarios, that can identify most of the patterns in the trajectories. However, it may or may not be applicable in more complex scenarios like the movement of trajectories in crowded environment, that sometimes depends on neighboring movements and so forth. In future, our effort would be to predict the basic attributes like latitude, longitude and timestamp of a GPS point, that can be georeferenced over the actual geographical map, and in the crowded environment.

## Figures and Tables

**Table 1:** Average Displacement Error (ADE) loss comparison of methods at different level of grid resolution (10 Pts).

Method/ADE	Grid Resolution			
	10 m	5 m	3 m	2 m
Linear Regression	9.37	13.07	21.22	27.03
Regression Tree	8.92	11.16	20.11	24.63
MLP	8.06	10.38	17.78	20.57
Simple RNN	6.18	9.73	14.43	16.14
CNN	4.72	7.05	9.24	10.71
GRU	4.07	5.49	7.69	8.36
Vanilla LSTM	4.18	5.61	7.46	8.17
LSTM (Enc/Dec)	4.05	5.58	7.34	7.93
ConvLSTM (Enc/Dec)	3.88	5.13	5.98	6.56
ConvLSTM (Enc/Dec) + Att	2.83	4.15	4.84	5.28
U-ConvLSTM (Enc/Dec) + Att + AuxGlobal	2.58	3.88	4.54	4.96
B-ConvLSTM (Enc/Dec) + Att + AuxGlobal	2.53	3.76	4.38	4.79

**Table 2:** ADE loss comparison of methods at different level of grid resolution (10 Pts).

Missing Length (No. of Points)	Grid Resolution			
	10 m	5 m	3 m	2 m
10 Pts	2.53	3.76	4.38	4.79
15 Pts	3.02	4.09	4.92	5.04
20 Pts	3.35	4.68	5.61	5.45
25 Pts	4.01	5.59	6.41	6.39
30 Pts	5.01	8.17	8.86	8.64
